# Maternal Modulation of Neonatal Immune System Development

**DOI:** 10.1080/10446670290030972

**Published:** 2002-03

**Authors:** Omar R. Fagoaga, Sandra L. Nehlsen-Cannarella

**Affiliations:** Department of Pathology, Immunology Center Loma Linda University School of Medicine and Medical Center 11234 Anderson St. Room 2578 Loma Linda CA 92354-2870 USA

## Abstract

Changes in programming of neonatal immune development were effected through maternal immune modulation (*Leishmania major inoculation*). In progeny of these dams, immune profiles in both blood and spleen were changed throughout the neonatal period and were pronounced after weaning. White blood cell (WBC) and lymphocyte counts in blood of 45-day-old progeny were two-fold less than control animals. In blood, proportions of B cells were greater, while T helpers, Tc/s and NK cells were less than in controls. In contrast, proportions of splenic B and NK cells were greater than controls. But, proportions of all T and Tc/s cells on d20 and 45 were lower than controls. In blood, absolute numbers of all T, Th naïve and Th memory cells were lower than in controls. In contrast, in the spleen, numbers of NK, T and Th naïve and memory cells were up to 200% greater than in control pups. Cytokine responses of splenic lymphocytes stimulated through CD3 ligation revealed no difference in IL-4 production. In contrast, IL-2 and IFNγ were lower on d45 and 5, respectively, in the experimental compared to control mice. These data support the hypothesis that maternal immune events during gestation can modulate the pattern of immune development in offspring.


*Developmental Immunology*, 2002 Vol. 9 (1), pp. 9–17

### Maternal Modulation of Neonatal Immune System Development

OMAR R. FAGOAGA and SANDRA L. NEHLSEN-CANNARELLA*

*Department of Pathology, Immunology Center, Loma Linda University School of Medicine and Medical Center, 11234 Anderson St. Room 2578, Loma Linda, CA 92354-2870 USA*

*(Submitted August 2000; Accepted 10 April 2001)*

Changes in programming of neonatal immune development were effected through maternal immune modulation (*Leishmania major* inoculation). In progeny of these dams, immune profiles in both blood and spleen were changed throughout the neonatal period and were pronounced after weaning. White blood cell (WBC) and lymphocyte counts in blood of 45-day-old progeny were two-fold less than control animals. In blood, proportions of B cells were greater, while T helpers, Tc/s and NK cells were less than in controls. In contrast, proportions of splenic B and NK cells were greater than controls. But, proportions of all T and Tc/s cells on d20 and 45 were lower than controls. In blood, absolute numbers of all T, Th naïve and Th memory cells were lower than in controls. In contrast, in the spleen, numbers of NK, T and Th naïve and memory cells were up to 200% greater than in control pups. Cytokine responses of splenic lymphocytes stimulated through CD3 ligation revealed no difference in IL-4 production. In contrast, IL-2 and IFNγ were lower on d45 and 5, respectively, in the experimental compared to control mice. These data support the hypothesis that maternal immune events during gestation can modulate the pattern of immune development in offspring.

### INTRODUCTION

Challenges of the maternal immune system during, and even before, pregnancy have been shown to be capable of modulating neonatal immune responses. Recent data suggest that this modulation is mediated by T helper cells and, depending on the antigen, may be expressed as a reduction in IgM and IgG responses to specific immunization in the 6-week-old pup or as an increase in IgM levels in fetal circulation (Fujii and Yamaguchi, 1992). The latter suggests an advancement in isotype switching in the neonate. Perhaps, this temporal advancement is the result of maternal factors crossing the placenta. Furthermore, not only humoral but also cell-mediated immunity has been modulated (Field and Caspary, 1971; Cramer *et al.*, 1974; Russell, 1975). These investigators reported neonatal immunity to antigens recognized by the maternal immune system before conception or during pregnancy and proposed such explanations as transplacental passage of antigen, transfer factor, antibodies and sensitized maternal cells leading to active sensitization of the neonate.

The mechanism for fetal sensitization has been proposed to be a result of either maternal cell passage, vertical antigen transmission or maternal transfer of soluble factors. Cell traffic across the placenta has been well documented in humans (Desai and Greger, 1963; Russell, 1975; Pollack *et al.*, 1982) and in the mice (Beer *et al.*, 1977; Piotrowski and Croy, 1996). Maternal lymphoid cells naturally enter the allogeneic fetus and take up residency in liver, spleen and bone marrow of wild type mice (Piotrowski and Croy, 1996). Passage of maternal cells into the fetus could explain the observed B cell unresponsiveness to non-inherited maternal HLA specificities documented in adults that are otherwise highly reactive to alloantigens (Claas *et al.*, 1988). Findings of these studies indicate that maternal cells, transiting the uteroplacental interface, could directly convey information to the fetus about pathogens in the environment.

Vertical transmission of antigens, however, appears to be a viable alternative explanation for fetal sensitization to maternal antigens (Stastny, 1965; Horton and Oppenheim, 1976). Still others (Barnetson *et al.*, 1976) have proposed the presence of a soluble lymphocyte factor by demonstration of sensitization to *Mycobacterium leprae* in newborn of infected mothers. On the other hand, maternally derived cytokines are potential candidates for modulating fetal and, subsequently, neonatal immunity.

*Corresponding author. Tel.: +1-313-966-0936. Fax: +1-313-966-0934. E-mail: snehlsen@dmc.org

ISSN 1044-6672 print © 2002 Taylor & Francis Ltd
DOI: 10.1080/10446670290030972

TABLE I Cytokine concentrations (mean ± SE) in serum (primary immunization) and from supernatants of spleen cell cultures (recall study) of *L. major*-immunized pregnant and non-pregnant CD-1 mice

| | Cytokine concentration (mean, pg/ml ± SE) | | | | | |
|---|---|---|---|---|---|---|
| | Serum | | | Supernatant | | |
| | IL-2 | IFNγ | IL-4 | IL-2 | IFNγ | IL-4 |
| Non-pregnant, saline ($n = 3$) | bas | 329 ± 57 | bas | 5 | bas | bas |
| Non-pregnant, *L. major* ($n = 4$) | bas | 486 ± 31 | bas | 210 ± 57 | 13 ± 13 | bas |
| Pregnant, saline ($n = 3$) | bas | 389 ± 70 | bas | Bas | 13 ± 13 | bas |
| Pregnant, *L. major* ($n = 3$) | bas | 379 ± 50 | bas | 145 ± 78 | 99 ± 44 | bas |

bas = Below assay sensitivity.

The placental barrier to cytokines appears to be selective. Granulocyte colony-stimulating factor readily passes the uteroplacental barrier (Medlock *et al.*, 1993) while others such as IL-8 (Reisenberger *et al.*, 1996) and erythropoietin (Koury *et al.*, 1988) do not. Although effects of transplacental passage of cytokines on fetal immune function are clearly possible, it is known that cytokines do not convey antigen-specific information that could direct antigen-specific perinatal host immunity. However, their transfer across the placenta raises the possibility that the normal fetal and postnatal immune development may be changed by maternal immune experiences during gestation.

Maturational profiles of immune phenotypes ($Ig^+B$, Thy-1, CD4, CD8 cells) during the neonatal period have, however, been reported to be seemingly preprogrammed (Fagoaga and Nehlsen-Cannarella, 2000). Furthermore, Harp and Sacco (1996) produced active infection (*Cryptosporidium parvum*) in young postnatal animals and observed unchanged patterns of immune cell development; on first examination, these findings seem to indicate the presence of an internal clock unaffected by postnatal events. To date, no evaluation of the impact of maternal immune experiences during gestation on immune development of cell phenotype and cytokine function in the progeny has been reported.

The postnatal immune system is defined by the profile of immune cell phenotypes and by their capacity to mount appropriate cytokine responses when challenged. We proposed that these responses could be modulated by maternal immune activity during gestation. More specifically, induction of maternal T helper 1 immune responses during gestation modulate patterns of fetal immune development (cell phenotypes and cytokine response capabilities). To test this hypothesis, pregnant dams received an injection of *Leishmania major* extract (protozoa killed by sonication), and the phenotypic profiles and cytokine response capabilities of their pups were studied. Specific sensitization of pups was tested *in vitro* using a "memory recall" protocol (spleen cells challenged with *L. major* extract *in vitro*) by assessing production of cytokines in culture after a brief incubation. It was anticipated that immune phenotypes and cytokine profiles of these pups would be modulated.

### RESULTS

Pregnant (gestational d13) CD-1 mice were inoculated with *L. major* extract (intraperitoneal). This immunization, designed to induce maternal inflammatory (Th1) immune responses, was tested by challenging maternal spleen lymphocytes (collected on the day of delivery) and assessing IFNγ and IL-2 production. Based on historical reports, it was hypothesized that progeny of these immunized dams would be sensitized to *L. major* antigen and have an advanced repertoire of immune cell phenotypes and Th1 functional capacity.

After 8 days of *L. major* inoculation (175 μg/animal), both IL-2 and IL-4 concentrations in serum from pregnant and non-pregnant controls were undetectable, Table I; while IFNγ, although measurable, was not different between groups. IL-2 concentrations in cell culture supernatants from pregnant animals were significantly ($t$ test, $p < 0.05$) increased in experimental compared to control animals. Similarly, IL-2 concentrations in non-pregnant animals were significantly higher ($p < 0.05$) than controls. In contrast to IL-2, IFNγ concentrations were not different between the cultures of controls and experimental animals. In culture supernatants, no IL-4 was detected in any group. This study confirmed that *in vivo* treatment with *L. major* extract could elicit a Th1 immune response (IL-2 and IFNγ) in both pregnant and non-pregnant CD-1 mice. It also confirmed that 16.6 μg *L. major* extract/well was sufficient antigen concentration for *in vitro* testing of sensitization.

### Ontogenic Development of Immunophenotypes in Progeny Born to Dams Inoculated with *Leishmania major*

White blood cell (WBC) and total lymphocyte counts in blood of 45-day-old experimental progeny were lower than those in control animals. In blood, the WBC on d20 was 1.5-fold greater than control cohorts (two-way ANOVA, interaction between factors, $p < 0.05$). However, by d45, WBC of experimental progeny were significantly lower (2-fold) from those of control progeny (Student's $t$-test, $p < 0.05$). Absolute numbers of lymphocytes in blood of experimental progeny were significantly lower only on postnatal d45 compared to controls (two-way ANOVA, interaction between factors, $p < 0.05$). In contrast to blood,

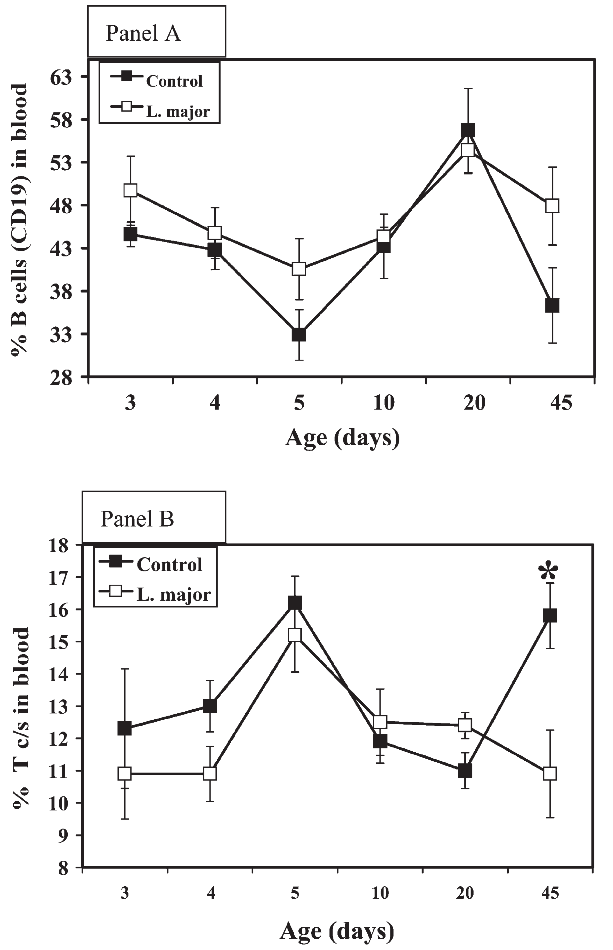

FIGURE 1 Proportions of B (CD19, Panel A) and Tc/s (CD8, Panel B) cells in blood of pups at various ages delivered from dams treated during gestation with saline or *L. major* (175 µg/mouse i.p. in saline). Data points are mean ± SE of 5 samples/age, except 6 for d20 saline group; and 5 samples/age for *L. major*. Samples represent pools of randomized pups (d3-10, 5–14/pool; d20, 2/pool; d45, 1 pup). Statistical significance ($p < 0.05$; $df = 1$; $F = 4.18$ for CD19; and $F = 4.13$ for CD8) for treatment effects among groups was derived from two-way ANOVA. *Post hoc* analyses were performed with Student's *t*-test to reveal differences among groups per age (*, $p < 0.05$). See "Statistical Analysis" section for further details. At some ages, symbols encompass area of SE bars.

in spleen, treatment effects were observed only in absolute numbers of lymphocytes; these were 1.3-fold higher on d45 than in control mice (Student's *t*-test, $p < 0.05$). Thus, treatment effects were observed but they were not apparent until after weaning. Proportions of lymphocyte subsets in offspring born to experimental or control dams were then compared for treatment effects.

### Proportions of Immune Cell Phenotypes in Blood

In blood of experimental progeny, proportions of B cells were greater (Fig. 1, panel A) while T cell subsets and NK cells were less than those in control progeny (Fig. 1, panel B). T cell effects were reflected in decreased Tc/s cells (two-way ANOVA, main effects, treatment vs. control, $p < 0.05$), that were lowest on d45 (Student's *t*-test, $p < 0.05$). Proportions of NK and naïve Th ($CD62^{pos}CD44^{low}$) in blood were significantly affected, changes were dependent on age [two-way ANOVA, interaction between factors (age vs. treatment), $p < 0.05$]. NK proportions in experimental animals were lower on d3 and higher on d10 (Student's *t*-test, $p < 0.05$), and tended (*t*-test, $p = 0.07$) to be higher on d45 when compared to control progeny of the same ages. In contrast, proportions of naïve Th were significantly lower on d4 (Student's *t*-test, $p < 0.05$) and tended ($p = 0.12$) to be higher on d45. Thus, *L. major* inoculation during pregnancy had a modulating effect on the proportions of specific immune cell phenotypes in circulation.

### Proportions of Immune Cell Phenotypes in Spleen

Proportions of splenic phenotypes in experimental progeny were increased compared to control progeny (Fig. 2). Main treatment effects were observed across all ages (two-way ANOVA, main effects, treatment vs. control group, $p < 0.05$) for CD19, CD3, CD4 and CD8. Overall, B cells were higher and T cells lower in experimental pups. Proportions of T cells on d20 and Tc/s cells on d20 and 45 were significantly below normal (Student's *t*-test, $p < 0.05$). NK cells were strongly affected by treatment, and were dependent on age (two-way ANOVA, interaction between factors, $p < 0.05$). On d10, the NK population was 1.7-fold higher than controls (Student's *t*-test, $p < 0.05$). In summary, proportions of B cells in blood and spleen were higher, while T cells were lower in experimental progeny compared to control progeny.

### Numbers of Immune Cell Phenotypes in Blood

Absolute numbers of blood cells in experimental progeny were lower than control progeny, including T, naïve Th ($CD62L^{pos}CD44^{low}$) and memory Th ($CD62L^{neg}$ $CD44^{high}$), (Fig. 3), and was the main treatment effect (two-way ANOVA, main effects, control vs. treatment group, $p < 0.05$). Furthermore, decreases in numbers of all subsets of T cells were dependent on age (two-way ANOVA, interaction between factors, $p < 0.05$). T cells and T cell subsets were 1.9- to 3.4-fold lower than controls on d45, but Tc/s rose 1.5-fold higher on d20 (Student's *t*-test, $p < 0.05$). In general, *L. major* inoculation during gestation decreased the number of blood immune cell phenotypes in progeny, but most of the effect was not apparent until adulthood. The specific time when the effects were more noticeable post-weaning could not be determined because data between d20 and 45 was not collected.

### Numbers of Immune Cell Phenotypes in Spleen

The principal treatment effects observed in spleen cells were higher NK and naïve Th cells (Fig. 4; two-way ANOVA, main effects, control vs. experimental progeny, $p < 0.05$). Additional effects of treatment, significantly

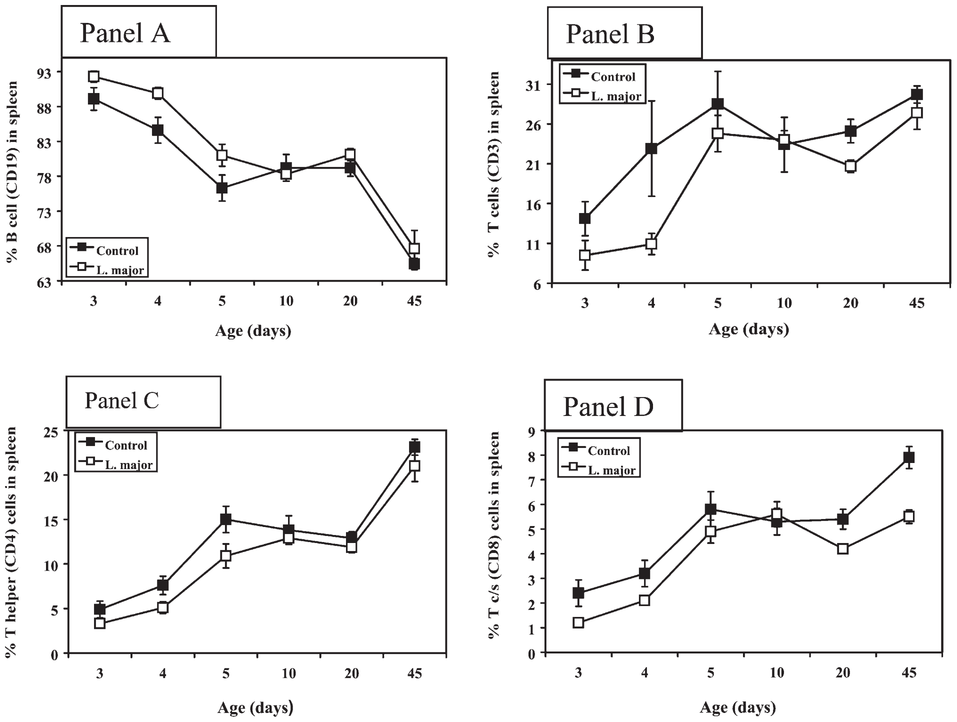

FIGURE 2 Main treatment effects were observed across all ages (two-way ANOVA, main effects, control vs. treatment group, $p < 0.05$) for proportions of B (CD19, Panel A), T (CD3, Panel B), Th (CD3CD4, Panel C) and Tc/s (CD3CD8, Panel D) in spleen of pups of various ages. Progeny was delivered from dams treated during gestation with saline or *L. major* (175 μg/mouse i.p in saline). Data points are mean ± SE of 5 samples/age. Samples represent pools of randomized pups (d3–10, 5–14/pool; d20, 2/pool; d45, 1 pup). Statistical significance ($p < 0.05$) for treatment effects among groups was derived from two-way ANOVA. These analyses generated $F$ statistics of 9.8 (CD19), 7.7 (CD3), 10.3 (CD4) and 16.4 (CD8) with 1 degree of freedom. No significant group interaction was observed for all. *Post hoc* analyses with Student's $t$-test revealed differences among groups per age: d4 (CD19), d20 (CD3 and CD8). See "Statistical analysis" section for further details. At some ages, symbol area encompasses SE bars.

dependent on age (two-way ANOVA, interaction between factors, $p < 0.05$), were greater numbers of NK, T and Th naïve and memory cells than found in control pups. Absolute numbers of naïve and NK cells on d45 were significantly higher (Student's $t$-test, $p < 0.05$), 140 and 200%, respectively, than those in control mice. Thus *L. major* treatment during pregnancy had an up-regulating effect on populations of T, naïve and memory Th and NK cells in spleens of progeny and this effect was pronounced at post-weaning age. Missing data (no assessment between d20 and 45) prevents accurate determination of when these changes actually manifest.

### Ontogenic Development of Cytokine Response Capabilities of the Progeny Born to *Leishmania major* Treated Dams

Functional capabilities of splenic lymphocytes were tested as previously described (CD3-ligated mixed cell cultures and cytokine levels in supernatants). IL-4 production did not differ between *L. major*- and saline-treated groups. In contrast, Th1 cytokines decreased in response to CD3 ligation. IL-2 was significantly lower ($p < 0.05$) on d45. The modulating effects of *L. major* inoculation on IFNγ production was associated with age (two-way ANOVA, interaction between factors, $p < 0.05$). *Post hoc* comparisons with Student's $t$-test revealed significantly (Fig. 5, Student's $t$-test, $p < 0.05$) lower IFNγ concentrations on d5. Thus, data of this study have documented that induction of immune reactivity in dams during gestation induces modulation in cytokine response capabilities of offspring.

### Evaluation of Sensitization to *Leishmania major* in Progeny

To determine whether progeny of inoculated dams had developed antigen-specific sensitization, spleen cells were challenged (72-h culture) with *L. major* extract (17 μg). Supernatants were then analyzed for cytokines (IL-4, IL-2 and IFNγ). No cytokines were detected in progeny from saline or *L. major* treated dams. These data indicate that the pups born to experimental dams may not have been sensitized to *L. major in utero*.

### DISCUSSION

In this study, changes in programming of neonatal immune development were effected through maternal immune modulation (response to *L. major* inoculation). In progeny of these dams, maturational profiles of immune cells in both blood and spleen were changed throughout the neonatal period, and these changes were more pronounced

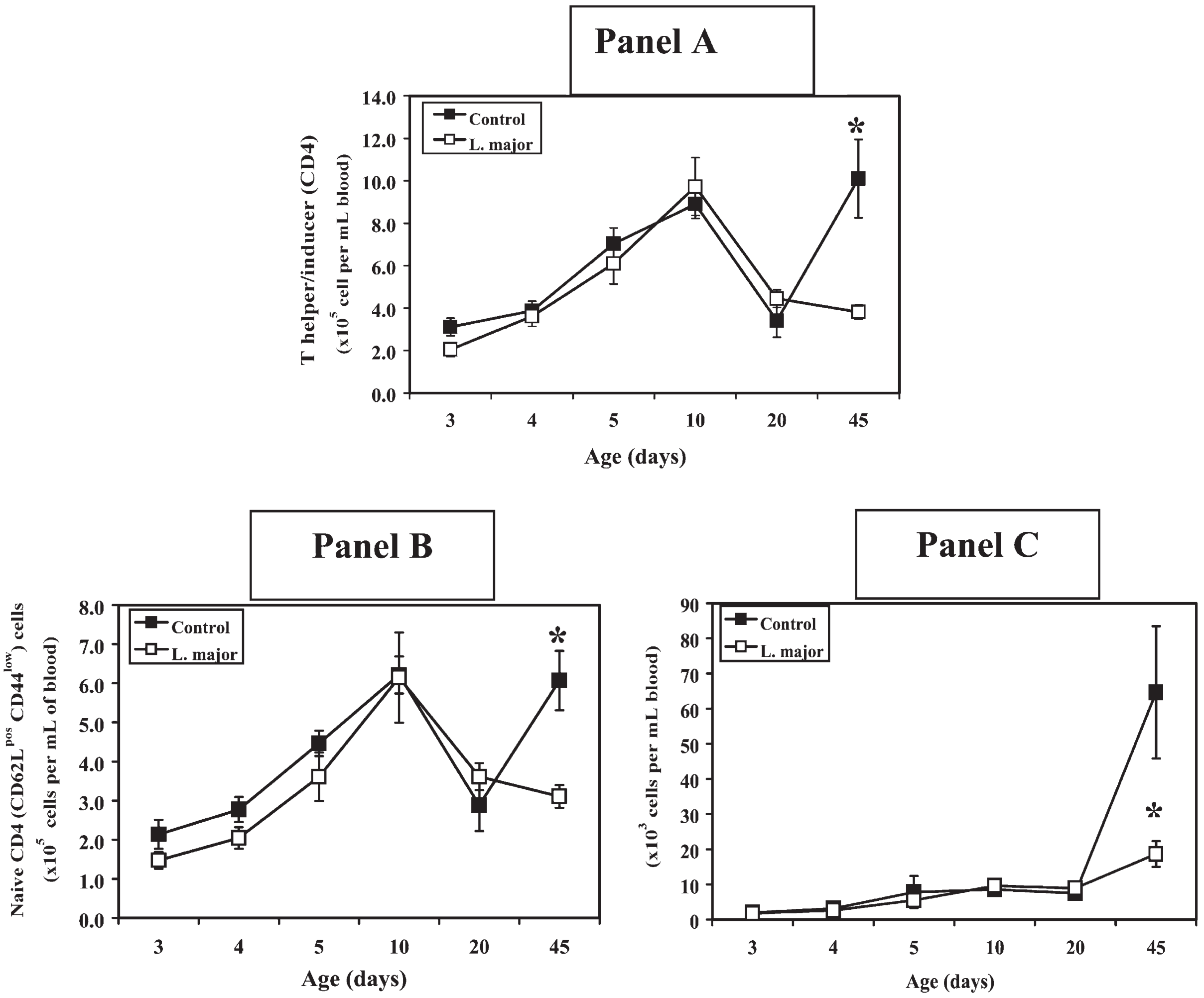

FIGURE 3 Absolute numbers of Th (CD4, Panel A), naïve Th ($CD62^{pos}CD44^{low}$, Panel B) and memory Th ($CD62L^{neg}CD44^{high}$, Panel C) cells in blood of pups of various ages delivered from dams treated during gestation with *L. major* (175 μg/mouse i.p.) or saline. Data points are mean ± SE of 5 samples/age, except 6 for d20 in saline group; and 5 sample/age in *L. major* group. Samples represent pools of randomized pups (d3-10, 5–14/pool; d20, 2/pool; d45, 1 pup). Statistical significance ($p < 0.05$; $df = 1$; $F = 4.9$ for Th; 2.4 for naïve and 5.3 for memory) for treatment effects among groups was derived from two-way ANOVA. Interaction between treatment and age suggest that effects are seen only on d45 ($F$ statistic: naïve = 2.4 and memory = 5.3; $df = 5$, $p < 0.05$). *Post hoc* analyses with Student's $t$-test (two-tail) revealed differences among groups per age (*, $p < 0.05$; $df = 8$; $t$ statistic of $-3.35$ (CD4), $-3.64$ (naïve), and $-3.64$ (memory). See "Statistical analysis" section for further details. At some ages, symbols obscure SE bars.

after weaning. Blood leukocytes, lymphocytes and specifically T cells of experimental progeny were lower than in control progeny and this difference was noted to be marked on d45. In contrast, spleen lymphocytes, T cells, naïve and memory Th cells and NK cells of experimental progeny were higher on d45 compared to control progeny, suggesting an increased homing of circulating lymphocytes to splenic compartments. That effects of maternal immune experience during gestation are most apparent in progeny reaching adulthood, may be a result of yet further maternal-mediated immune regulation, perhaps through passive transfer of immunoglobulins during late gestation and via milk during the suckling period (Newman, 1995). Some of these regulatory products in milk include antibodies, cells (lymphocytes, macrophages and neutrophils), lactoferrin, B12 binding protein, bifidus factor, fibronectin, gamma-interferon, hormones, growth factors and lysozyme.

Expectations were that this experiment would result in up-regulation of Th1 function; instead, it appears that Th2 functions may have been induced. Spleen cells of progeny had decreased production of IL-2 and IFNγ compared to normal controls. It is possible that a state of tolerance rather than inflammatory reactivity was induced in dams and pups, evidenced by the decrease in Th1 cytokine production in response to polyclonal stimulation. The decrease in Th1 cytokine production can be explained by taking into account three facts: soluble antigens primarily induce Th2 reactions (Zinkernagel and Kelly, 1997); inoculation in mid-gestation can lead to tolerance (Beer *et al.*, 1977); and down-regulation of Th1-type cytokines is a consequence of tolerance induction (Chen and Field, 1995). If dams had been tolerized and this immune status passed to their progeny, a shift in cytokine profile away from Th1 would be expected. Cytokines production might have been modified by the abundant immune products that

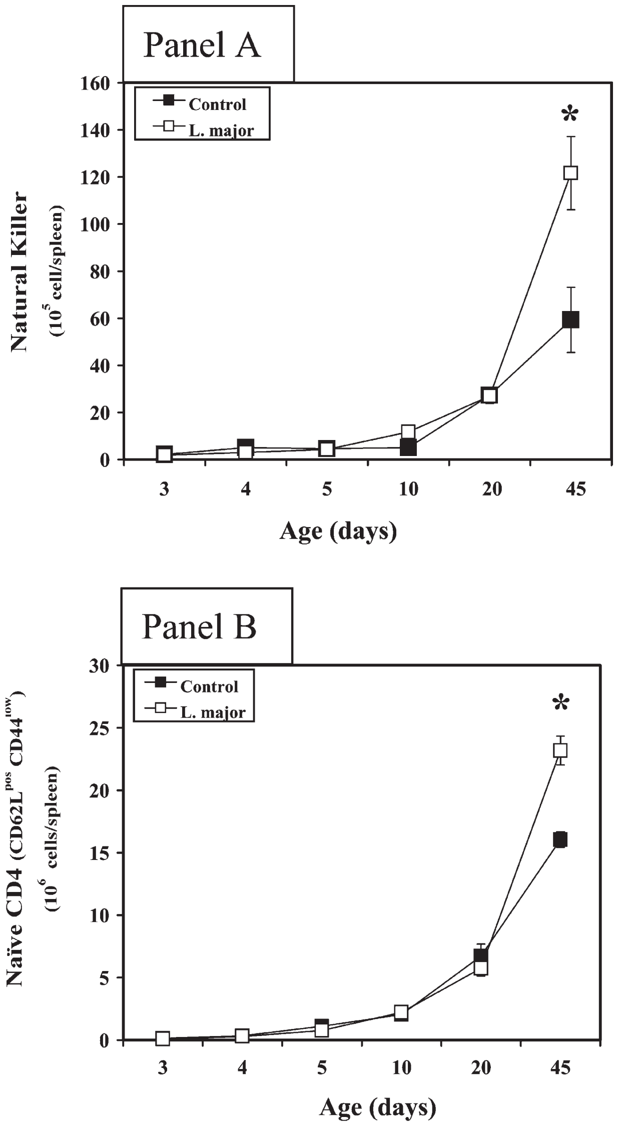

FIGURE 4 Natural killer (NK, Panel A) and naive Th cells (CD62LposCD44low, Panel B) in spleens of pups of various ages delivered from dams treated during gestation with saline or *L. major* (175 ug/mouse i.p. in saline). Data points are mean ± SE of 5 samples/age, except 6 on d20 in saline group; and 5 sample/age for *L. major*. Samples represent pools of randomized pups (d3-10, 5–14/pool; d20, 2/pool; d45, 1 pup). Statistical significance ($p < 0.05$; $df = 5$; $F = 8.27$ for NK and 16.2 for naive) for treatment effects among groups was derived from two-way ANOVA. *Post hoc* analysis with Student's *t*-test (two-tail) to reveal differences among groups per age (*, $p < 0.05$; $df = 8$; *t* statistic of 2.99 (NK) and 5.4 (naïve)). See "Statistical analysis" section for further details. At some ages, symbols obscure SE bars.

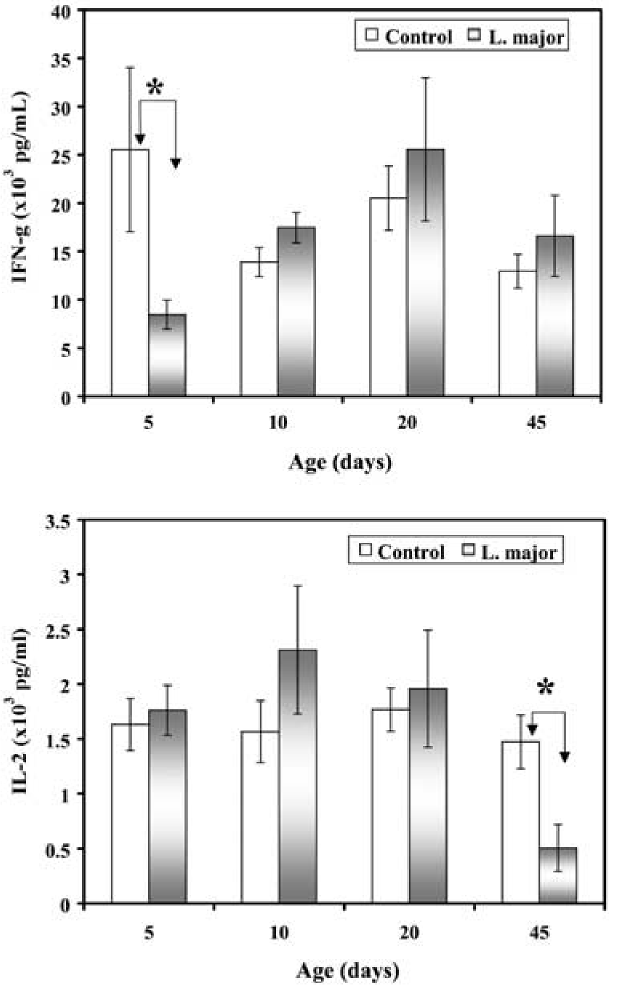

FIGURE 5 IFNγ (top panel) and IL-2 (bottom panel) production (stimulated) by spleen cells of CD-1 outbred mice (male and female) delivered from dams treated during gestation with saline or *L. major* (175 μg/mouse i.p in saline). Each culture contained $1 \times 10^6$ CD3 + CD4 + splenocytes and hamster anti-mouse CD3 monoclonal antibody in complete media. Control cells were cultured in media only. Data are expressed as mean ± SE of samples (control: d5 = 4, d10 = 3, d20 = 10, d45 = 11; experimental: d5 = 7, d10 = 7, d20 = 3, d45 = 5). Samples in stimulated cultures were pools of randomized pups (d5 and 10, 10/pool; d20, 3/pool; d45, 1 pup). Statistical significance ($p < 0.05$; $df = 3$; $F = 3.6$) between groups was derived from two-way ANOVA. *Post hoc* analyses with Student's *t*-test (two-tail) revealed differences among groups per age (*, $p < 0.05$; $df = 9$ (IFNγ) and 14 (IL-2); *t* statistic of −2.63 for IFNγ and −2.45 for IL-2). See "Statistical analysis" section for further details.

are delivered through suckling (Newman, 1995), thus, explaining the observation of more significant differences being noted post-weaning. However, this conclusion cannot be validated with the current data and further experimentation is required to explore the influence of milk immune products acquired through suckling in this model.

The present study raises the possibility that maternal immune experiences may equip offspring *in utero* to defend against specific pathogens encountered outside the womb while preventing vertical transmission of infection. If this had been the case, sensitization of experimental offspring to *L. major* antigen would have been induced. The memory recall experiments, performed by challenging spleen cells of offspring with *L. major* extract, produced no detectable cytokines after three days of culture. These results indicate lack of sensitization to *L. major* in the offspring. Alternatively, absence of response may be due to challenging neonatal cells with too high antigen concentration; it has been reported by one investigative team that neonatal antigen presentation is 100-fold more efficient than adults (Van Tol *et al.*, 1983; 1984). The dose of *L. major* antigen used in the memory recall assays had been optimized with adult splenocytes, a deficiency in this study that needs further investigation. However, assuming that a prozone effect had not occurred, these results were unexpected since Herman *et al.* (1982) reported sensitization of offspring from *Leishmania donovani*-infected hamsters. Lack of sensitization in our experimental progeny may also be the result of using a non-pathogenic, purified extract administered i.p.; Herman *et al.* (1982) administered live amastigotes (active intracardiac infection) to their experimental animals.

Active infection is processed differently than an injection of soluble antigen from a killed parasite. The life cycle of this parasite is complex and during the infection process, promastigotes express abundant gp63 glycoprotein. On the other hand, amastigotes (from which the extract was made) express very low levels of this protein (Kurtzhals *et al.*, 1994; Reiner and Locksley, 1995). The cytokine environment during induction of immune responses to gp63 determines the course of disease resulting from the type of immune responses induced (Kurtzhals *et al.*, 1994; Reiner and Locksley, 1995). Humoral responses are primarily induced by this protein (Kurtzhals *et al.*, 1994) in the early phase of infection and Kemp *et al.* (1994) showed that cytokine response in visceral Leishmaniasis is primarily a Th2 response, whereas cutaneous Leishmaniasis elicits predominantly Th1 response. This suggests that there are different cytokine environments in visceral vs. cutaneous sites, and that response is also dependent on route of administration (i.p. vs. subcutaneous).

The unexpected result of these experiments may be rooted not only in the type of antigen used to induce Th responses during pregnancy, but also the immunization program (dose, route and timing; Zinkernagel and Kelly, 1997). In this work, *L. major* extract was injected i.p. and this may be a factor for lack of sensitization in progeny, even though maternal spleens tested *in vitro* tended to a polarized Th1 response. Time of inoculation during pregnancy may be another factor; in a different model, tolerization resulted when dams were inoculated 5–7 days antepartum (Beer *et al.*, 1977). In the current work, *L. major* extract was given 7 days antepartum, a time chosen as most likely time to avoid fetal loss due to release of Th1 cytokines (Krishnan *et al.*, 1996). This single immunizing event may not have been sufficient to induce sensitization in the offspring, and instead may have induced tolerance. Sustained chronic immune responses to live *L. donovani* over the whole course of pregnancy could explain sensitizations realized by Herman *et al.* (1982).

In summary, the data indicate that at maturity, T cells in blood augmented their homing into lymphoid compartments as evidenced by significant increases in splenic NK and T cell numbers at postnatal d45 (Fig. 4). Furthermore, these changes in splenic cellularity produced significant decreases in IL-2 (d45) and IFN$\gamma$ (d5) production by activated spleen cells compared to controls. Taken together, these data support the hypothesis that immune events experienced by the mother during gestation can modulate the pattern of immune development (cell phenotypes and cytokine response capabilities) in the offspring. Although significant effects in development were induced during gestation, a complete understanding of the mechanism must be ascertained before elective manipulation can be entertained. Timing and mode of delivering sensitizing or tolerizing immunogens must be defined, as well as dose and schedule of immunization. Of considerable importance is the need to determine what effect concurrent immune events would have on the intended process since regulatory function promulgated by APC is subject to prevailing signals dictated by local neuroendocrine immune products.

### MATERIALS AND METHODS

CD-1 mice, a genetically wild-type strain were purchased from Charles River Laboratories (Wilmington, MA). The first pregnant CD-1 mice arrived in the vivarium on gestational d13. All mice were housed individually in small cages in the same room and maintained in a 12- and 12-hour light–dark cycle. Postpartum females, adult males and progeny provided the breeding nucleus needed to generate additional pups for later studies. A "trios" method of breeding was employed. Briefly, one male was mated to two females and housed together for 48 h. Females were then individually housed; a vaginal plug confirmed that copulation had occurred. The limited duration of conspecific exposure coordinated the timing of birth. Body weights of female breeders were measured daily to confirm the successful progress of pregnancy.

### Collection of Blood and Spleen

Mice, 10 days of age or less were killed by decapitation and blood was collected into heparinized capillary tubes from cut surface of the body. For 20- and 45-days old groups, blood was collected from individual mice by cardiac puncture following pentobarbital anesthesia (40 mg/kg body weight); then mice were killed by decapitation. The spleen was extracted from the peritoneal cavity of each mouse under sterile procedures, weighed without mesenteric fat, and placed in sterile RPMI-1640 media (Fisher Scientific, Pittsburgh, PA) at room temperature. Lymphocytes were harvested within 30 min. Briefly, a spleen cell suspension was prepared by gently pressing tissue through a fine nylon mesh sieve and resuspending in complete media (RPMI-1640 containing prescreened 10% fetal calf serum and 1% penicillin/streptomycin). These reagents were purchased from Fisher Scientific, Pittsburgh, PA. Leukocytes in all blood and spleen cell suspensions were counted with a Unopette blood counting system (Becton Dickinson, Franklin Lakes, NJ) and a hemocytometer.

Pools were created by mixing blood or spleens of randomly-sorted pups from different litters of the same age. Each pool contained eight pups each for ages 0, 1 and 2 days. Although adequate sample volumes were obtained for immunophenotyping, it was impractical to perform experiments for study of cytokine production in mice before 2 days of age.

### Immunophenotyping

Flow cytometric analysis was performed as previously described (Fagoaga *et al.*, 2000). Briefly, single cell preparations from whole blood and spleen ($1 \times 10^5$ cells/tube) were stained with 10 μl fluorochrome-conjugated monoclonal antibodies (mAb). A mAb panel was constructed for two- (fluoroisothiocyanate [FITC] and phycoerythrin [PE]) and three-color immunophenotyping (FITC, PE and Cy-Chrome). Two-color analysis was used to enumerate B vs. T cells (CD19*FITC/CD3*PE), T helper/inducer cells (CD4*FITC/CD3*PE), T cytotoxic/-suppressor cells (CD8*FITC/CD3*PE) and natural killer cells (CD3*FITC/NK1.1*PE). Three-color analysis was used to assess naïve and memory T helper cells (CD62L*FITC/CD44*PE/CD4*Cy-Chrome). All rat anti-mouse mAb were obtained from PharMingen (San Diego, CA), except CD3*FITC (hamster anti-mouse antibody) obtained from Caltag Laboratories (San Francisco, CA). After incubation (15 min, 4°C), erythrocytes were lysed with 0.8% ammonium chloride solution and samples centrifuged at 300*g* for 5 min. Each supernatant was aspirated and cell pellets resuspended in 300 μl phosphate buffered saline (PBS).

Analytical controls included (a) unstained cells and (b) cells mixed with isotype antibody to evaluate degree of non-specific staining. Furthermore, an unlabeled, purified rat anti-mouse CD16/CD32 (FcγIII/II receptor; PharMingen, San Diego, CA) monoclonal antibody was used as an agent for blocking non-specific binding through the FcγIII/II receptor.

Proportions of lymphocyte populations (CD3, CD3CD4, CD3CD8, NK, CD19) were expressed as percentages normalized to lymphocyte purity (sum of lymphocyte subsets; CD19 + CD4 + CD8 + NK). This normalization excluded debris, other non-lymphoid cells (mainly monocytes) and Tγδ cells ($CD3^{pos}CD4^{neg}$ $CD8^{neg}$). Normalization was not required for analyzing T helper cells for CD62L and CD44 expression because these cells were selectively gated, based on their far red (CyChrome) fluorochrome characteristic. Absolute numbers of each population were generated by multiplying WBC counts by the sum of proportions of lymphocyte subsets (i.e. $\sum CD19 + CD3 + NKs$) and again by proportion of each population (CD3, CD3CD4, CD3CD8, NK and CD19 subsets). Absolute numbers of T helper cells analyzed for expression of CD62L and CD44 were similarly generated.

### T Cell Stimulation Assay

A monoclonal antibody to CD3 was produced from cell culture of clone 145-2C11 (PharMingen, San Diego, CA) as previously described (Fagoaga and Nehlsen-Cannarella, 2000). Splenocytes were collected from spleens as described above. Subsequently, cell cultures were set up in 24-well plates (Fisher Scientific, Pittsburgh, PA). Cultures consisted of either unsorted single cell spleen suspensions (SSCS). To obtain a constant number of CD4 cells in each culture, absolute numbers of CD4 cells in unsorted cell preparations were determined by flow cytometry. An aliquot containing $1 \times 10^6$ CD3 + CD4 + splenocytes was added to each well with 1 ml of optimally diluted CD3 monoclonal antibody. Each culture volume was brought up to 2 ml with complete media.

### Cytokine Assays

Commercial cytokine ELISA kits from ENDOGEN (Woburn, MA) were used to assess IL-2, IL-4 and IFNγ production by murine splenocytes after CD3 ligation and after *in vivo* and *in vitro L. major* challenge. Assay procedures followed manufacture's instructions and as previously described (Fagoaga and Nehlsen-Cannarella, 2000).

### Determination of *In Vivo* and *In Vitro Leishmania major* Dose

*L. major* extract of killed protozoa (sonicated) (Antibody Systems, Hurst, TX) was suspended in saline (1 mg/ml). A pilot study was conducted with pregnant and non-pregnant ($n = 6$) mice to determine optimal *in vivo* dose to induce a Th1 response (IL-2 and IFNγ production) Table I. Each animal received 175 μl (intraperitoneal injection) of either *L. major* extract (pregnant, $n = 3$; control, $n = 3$) or saline (pregnant, $n = 3$; control, $n = 3$). This dose was recommended by Antibody Systems for Th1 induction. Pregnant animals were inoculated once (gestational d8, 11 or 13); no further injections were given as a precaution to avoid induction of premature labor and delivery. Spleens and sera were collected from dams on day of parturition (gestational d20) and from non-pregnant controls 9 days post-inoculation.

Successful immunization of these animals was confirmed by assessing cytokine concentrations (IL-2, IFNγ, IL-4) in serum and in supernatants from spleen cells in culture after *in vitro* challenge with *L. major* (recall). Sera for these determinations were obtained by cardiac exsanguination under sodium pentobarbital anesthesia as previously described. For the immune memory recall study, spleens were harvested from exsanguinated mice and single cell suspensions prepared as described. Cells were incubated ($1 \times 10^6$ cells/well) in 96-well microculture plates (Fisher Scientific, Pittsburgh, PA) with 16.6 μg/well of *L. major* extract ("starting" concentration suggested by supplier) in complete media for 72 h in 5% $CO_2$ in humidified air at 37°C. At the end of incubation, cell culture supernatants were collected and assayed for IL-2, IFN$\gamma$ and IL-4.

### Statistical Analysis

One-way ANOVA and Duncan's *post hoc* test for multiple comparisons among different ages were calculated. If test for homosedasticity was significant (greater than the critical value for $F$-max test for homogeneity of variances), data were log transformed. After transformation, if significant homogeneity of variances persisted, a Kruskal Wallis ANOVA with multiple comparisons was performed to assess differences among age groups. Two-way ANOVA was used to determine treatment effects of *L. major* extract inoculations. Furthermore, Duncan's test for multiple comparisons and $t$-tests were used as *post hoc* tests to determine group differences. A value of $p < 0.05$ was considered significant.

### *References*

Barnetson, R.S.T.C., Bjune, G. and Duncan, M.E. (1976) "Evidence for a soluble lymphocyte factor in the transplacental transmission of T-lymphocyte responses to *Mycobacterium leprae*", *Nature* **260**, 150–151.

Beer, A.E., Billingham, R.E., Head, J.R. and Parmely, M.J. (1977) "Possible influence of maternal-to-perinatal cell transfer on the development of host defenses", In: Cooper, M.D. and Dayton, D.H., eds, *Development of Host Defenses* (Raven Press, New York), pp 251–271.

Chen, N. and Field, E.H. (1995) "Enhanced type 2 and diminished type 1 cytokines in neonatal tolerance", *Transplantation* **59**, 933–941.

Claas, F.H., Gijbels, Y., van-der-Velden-de-Munck, J. and van-Rood, J.J. (1988) "Induction of B cell unresponsiveness to noninherited maternal HLA antigens during fetal life", *Science* **241**, 1815–1817.

Cramer, D.V., Kunz, H.W. and Gill, T.J. (1974) "Immunologic sensitization prior to birth", *Am. J. Obstet. Gynecol.* **120**, 431–439.

Desai, R.G. and Greger, W.P. (1963) "Maternofetal passage of leukocytes and platelets in man", *Blood* **21**, 665–673.

Fagoaga, O.R. and Nehlsen-Cannarella, S.L. (2000) "Ontogenic development of Th1 and Th2 function in the mouse", *J. Dev. Immunol.*, **8**(1), 47–60.

Fagoaga, O.R., Yellon, S.M. and Nehlsen-Cannarella, S.L. (2000) "Maturation of lymphocytes immunophenotypes and memory T helper cell differentiation during development in mice", *J. Dev. Immunol.*, In press.

Field, E.J. and Caspary, E.A. (1971) "Is maternal lymphocyte sensitization passed to the child?", *Lancet*, 337–342.

Fujii, Y. and Yamaguchi, N. (1992) "Maternal T cells of immunized pregnant mice induce immune suppression in their offspring", *Immunology* **77**, 171–176.

Harp, J.A. and Sacco, R. (1996) "Development of cellular immune functions in neonatal to weanling mice: relationship to *Cryptosporidium parvum* infection", *J. Parasitol.* **82**, 245–249.

Herman, R., Nolan, T.J. and Fahey, J.R. (1982) "Sensitization of offspring of *Leishmania donovani*-infected hamsters to immunization and of offspring of immunized hamsters to challenge", *Am. J. Trop. Med. Hyg.* **31**, 730–739.

Horton, J.E. and Oppenheim, J.J. (1976) "Relationship of transformation of newborn human lymphocytes by dental plaque antigen to the degree of maternal periodontal disease", *Cell. Imunol.* **21**, 153–160.

Kemp, M., Hey, A.S. and Kurtzhals, J.A.L. (1994) "Dichotomy of the human T-cell response to leishmania antigen. I. Th1-like response to *Leishmania major* promastigote antigens in individuals recovered from cutaneous leishmaniasis", *Clin. Exp. Immunol.* **96**, 410–415.

Koury, M.J., Bondurant, M.C., Graber, S.E. and Sawyer, S.T. (1988) "Erythropoietin messenger RNA levels in developing mice and transfer of 125I-erythropoietein by the placenta", *J. Clin. Investig.* **82**, 154–161.

Krishnan, L., Guilbert, L.J., Wegmann, T.G., Belosevic, M. and Mosmann, T.R. (1996) "T helper 1 response against *Leishmania major* in pregnant C57BL/6 mice increases implantation failure and fetal resorptions. Correlation with increased IFN-gamma and TNF and reduced IL-10 production by placental cells", *J. Immunol.* **156**, 653–662.

Kurtzhals, J.A.L., Hey, A.S., Jardim, A., Kemp, M., Schaefer, K.-U., Odera, E.O., Christensen, C.B.V., Githure, J.I., Olafson, R.W., Theander, T.G. and Khrazmi, A. (1994) "Dichotomy of the human T cell response to Leishmania antigens. II. Absent Th2-like response to gp63 and Th1-like response to lipophosphoglycan-associated protein in cells from cured visceral leishmaniasis patients", *Clin. Exp. Immunol.* **96**, 416–421.

Medlock, E.S., Kaplan, D.L., Cecchini, M., Ulich, T.R., del Castillo, J. and Andresen, J. (1993) "Granulocyte colony-stimulating factor crosses the placenta and stimulates fetal rat granulopoiesis", *Blood* **81**, 916–922.

Newman, J. (1995) "How breast milk protects newborns", *Sci. Am.* (December), 76–79.

Piotrowski, P. and Croy, B.A. (1996) "Maternal cells are widely distributed in the murine fetuses *in utero*", *Biol. Reprod.* **54**, 1103–1110.

Pollack, M.S., Kirkpatrick, D., Kapoor, N., Dupont, B. and O'Reilly, R.J. (1982) "Identification by HLA typing of intrauterine-derived maternal T cells in four patients with severe combined immunodeficiency", *N. Engl. J. Med.* **307**, 662–666.

Reiner, S.L. and Locksley, R.M. (1995) "The regulation of immunity to *Leishmania major*", *Ann. Rev. Immunol.* **13**, 151–177.

Reisenberger, K., Egater, C., Volg, S., Sternberger, B., Kiss, H. and Husslein, P. (1996) "The transfer of Interleukin-8 across the human placenta perfuded *in vitro*", *Obstet. Gynecol.* **87**, 613–616.

Russell, A.S. (1975) "Cell-mediated immunity to microbial antigens in mother and child", *Clin. Exp. Immunol.* **22**, 457–460.

Stastny, P. (1965) "Accelerated graft rejection in the offspring of immunized mothers", *J. Immunol.* **95**, 929–936.

Van Tol, M.J.D., Zijlstra, J., Heijnen, C.J., Kuis, W., Zegers, B.J.M. and Ballieux, R.E. (1983) "Antigen-specific plaque-forming cell response of human cord blood lymphocytes after *in vitro* stimulation by T cell-dependent antigens", *Eur. J. Immunol.* **13**, 390–397.

Van Tol, M.J.D., Zijlstra, J., Zegers, B.J.M. and Ballieux, R.E. (1984) "Distinct role of neonatal and adult monocytes in the regulation of *in vitro* antigen-induced plaque-forming cell response in man", *J. Immunol.* **134**, 1902–1908.

Zinkernagel, R.M. and Kelly, J. (1997) "How antigen influences immunity", *Immunologist* **5/4**, 114–120.

